# Efficient and fast arsenate removal from water by *in-situ* formed magnesium hydroxide

**DOI:** 10.1038/s41598-024-72258-6

**Published:** 2024-09-11

**Authors:** Juanjuan Zhou, Ying Yang, Zhanjun Li

**Affiliations:** 1 School of Health, Guangzhou Vocational University of Science and Technology, Guangzhou, 510080 People’s Republic of China; 2 School of Environment, Jinan University, Guangzhou, 511436 People’s Republic of China; 3 School of Biomedical Engineering, Guangzhou Medical University, Guangzhou, 511436 People’s Republic of China

**Keywords:** Arsenate, In-situ, Magnesium hydroxide, Removal, Environmental chemistry, Natural hazards

## Abstract

MgO nanoparticles have good As-adsorption capacity in treating As-contaminated wastewater but suffer from high production cost. In this study, instead of using pre-formed MgO nanoparticles, we found that in-situ formed Mg(OH)_2_ from MgCl_2_ and NaOH reaction exhibited super high arsenate (As(V)) removal efficiency. Only 1.5 mmol/L of in-situ formed Mg(OH)_2_ could remove more than 95% As(V) within 10 min to make the As contaminated water (10 mg-As(V)/L) meet the municipal wastewater treatment standard, whereas MgO nanoparticles failed. The Mg-As sludge has an amorphous crystal structure while no Mg(OH)_2_ phase could be observed. As(V) existed uniformly within the sludge which was confirmed by elemental mapping. A precipitation-adsorption-coagulation mechanism might exist, which could relieve the restriction of limited surface area of solid MgO adsorbents. This study not only reveals an applicable method for efficient removal of trace level As(V) from water but also implies the huge potential of in-situ formed adsorbents in water treatment.

## Introduction

Arsenic (As) is a Class I carcinogen due to its high toxicity to organisms^1,2^. In the past few decades, millions of people around the world suffered from various chronic diseases related to regular consumption of As contaminated water^3,4^. It has become a prerequisite to remove As from wastewater to prevent As contamination to our ecosystems. To date, many advanced technologies have been developed for this purpose, such as membrane separation, ion exchange, electrokinetics, coagulation and adsorption^5–7^. Coagulation and adsorption are well-studied strategies in water treatment due to their simplicity and cost-effectiveness^8,9^. A lot of coagulants/adsorbents have been reported with good As removal performance, such as oxides or hydroxides of aluminum (Al), iron (Fe), manganese (Mn), magnesium (Mg), zinc (Zn), titanium (Ti), zirconium (Zr), lanthanum (La), cerium (Ce), and yttrium (Y)^8–13^. However, existing adsorbents usually have low adsorption capacities at low As concentration, which leads to large amount of As-containing hazardous sludge that needs further disposal^8,9^. Therefore, there is still a great need to develop more efficient As-removal reagents and related technologies.

MgO is an eco-friendly material with low cost^14^. At present, MgO-based materials have been widely studied for water As removal, but most of them focus on MgO-based composites, such as Mg–Al layered double hydroxides^15,16^, zinc-magnesium oxides (ZnO-MgO)-based materials^17^, Mg-Fe-based hydrotalcite and oxides^18–20^. Although these MgO-based hybrid materials exhibited good performance on As removal, their complicated synthesis process will lead to increasing production cost and hinder their large-scale practical application. In fact, pure MgO or Mg(OH)_2_ itself already possesses high adsorption capacity to As^21^. MgO nanoparticles were reported with a maximum As adsorption capacity up to 115.27 mg/g^10^. In another study, nest-like micro-/nanostructured N-MgO was developed with a maximum adsorption capacity of 378.79 mg-As(V)/g-MgO, which was much higher than other micro-/nanostructured metal oxides^22^. Mechanism analyses indicated that As species were all removed by Mg(OH)_2_ because MgO hydrolyzed quickly forming Mg(OH)_2_ after it was added into water^22,23^. Because the *in-situ* formed Mg(OH)_2_ from MgO hydrolysis had more high affinity surface hydroxyl groups than pre-formed Mg(OH)_2_. Thus, generally, MgO performed better than Mg(OH)_2_ on As removal. Nevertheless, the cost of MgO, especially nanoparticles, is still too high for scale application in water treatment. Considering low material cost, MgCl_2_, a byproduct of salt industry and raw chemical for MgO production, is the cheapest Mg-related reagents. It might be much more cost-effective to use MgCl_2_ instead of MgO for water As removal. However, researches on using MgCl_2_ as As-removal reagents are still rare to date.

*In-situ* formed metal hydroxides were found more effective to adsorb pollutants than there pre-formed counterparts^24–26^. Therefore, we hypothesize that *in-situ* formed Mg(OH)_2_, using MgCl_2_ as Mg^2+^ source, might perform better than nano-MgO on water As removal. To verify this hypothesis, a simple comparison was conducted between MgO nanoparticles and *in-situ* formed Mg(OH)_2_ (by adding MgCl_2_ and NaOH) to reveal the advantages of As removal efficiency and kinetics of *in-situ* formed Mg(OH)_2_. Arsenate (As(V)) was used to simulate As-contaminaed wastewater because it is usually the most abundant As specie in surface water and arsenite (As(III)) is ease to be transformed into As(V) through an oxidation pre-treatment^5–7^. Detailed mechanism analysis was performed based on multiple analysis of the formed Mg-As sludge.

## Experimental

### Synthesis of nano-MgO

All reagents in this study were analytical grade and used directly without any further purification. Nano-MgO was synthesized following our previous report^27^. Briefly, 100 mL of Mg^2+^ solution (1 mol/L) was prepared by dissolving magnesium chloride in distilled water. Then, 15 mL of ammonium water (28 wt.%) was added quickly into Mg^2+^ solution with vigorous stirring to form Mg(OH)_2_. After boiling to let water evaporate, the obtained Mg(OH)_2_/NH_4_Cl mixture was then annealed at 450 °C for 2 h. Finally, loose white powder, nano-MgO, was obtained after simple grinding. The specific surface area and mesopore volume of synthesized nano-MgO were 66.83 m^2^/g and 0.135 cm^3^/g, respectively. They were acquired from the N_2_ adsorption/desorption isotherm which was measured using an automated gas sorption analyzer (Autosorb, Quantachrome, USA)^27^.

### Batch experiments

All batch experiments were conducted in a glass reactor contained 200 mL of 10 mg/L inorganic As(V) (Na_2_HAsO_4_·7H_2_O, Sigma-Aldrich) solution which was stirred by a magnetic stirrer. As(V) was chosen as the model As species because As(V) is the major component and the most stable form of As in aquatic environments^28^. For nano-MgO treatment, nano-MgO powder was added to each reactor to desired concentrations under stirring (~ 150 rpm). For *in-situ* formed Mg(OH)_2_ treatment, 0.1 mol/L MgCl_2_ and NaOH solutions were added successively drop by drop to each reactor to desired concentration. Specifically, in As(V) removal experiment, there were 5 application dosages (0.5, 1.0, 1.5, 2.0, and 2.5 mmol/L) for both nano-MgO and *in-situ* formed Mg(OH)_2_. All glass reactors were sealed and stirred (~ 150 rpm) at 25 °C for 24 h. For adsorption kinetics, 2 mmol/L of material dosage was chosen based on the As(V) removal experiment, and samples were collected at desired time intervals.

The effect of pH on As(V) removal by *in-situ* formed Mg(OH)_2_ was explored with pH values ranged from 10.0 to 11.5. The pH range started at 10.0 because the pH of solution is ~ 10 when Mg^2+^ and OH^−^ react at molar ratio of 1:2 to form Mg(OH)_2_. Initial pH was adjusted by 0.1 mol/L NaOH, and the pH at the end of experiment was also tested. The effect of co-existed ions and humic acid (HA) were conducted with co-existed ions (Na^+^, SO_4_^2−^, Ca^2+^, PO_4_^3−^, and CO_3_^2−^) of 10 mg/L or HA of 100 mg/L. All experiments were conducted at 25 ± 1 °C, except for the reaction temperature experiment, which was conducted at temperatures ranging from 25 to 40 °C.

There were three replicates for each experiment. At the end or desired time intervals, approximately 4 mL aqueous sample was collected and filtered through a 0.22 μm membrane filter. The concentration of residual As(V) was analyzed using a liquid chromatography-atomic fluorescence spectrometry (LC-AFS) (ELSPE-2, Guangzhou Pulin Sheng Technology Co., Ltd, China).

### Characterization

To further explore the underlying mechanism, the precipitates were collected via centrifugation after coagulation, washed with distilled water and dried at 80 °C. Fourier transform infrared (FTIR) spectra of samples were recorded with KBr pellets in the range of 4000–400 cm^−1^ using a Thermo Scientific Nicolet 6700 spectrometer. The morphologies of adsorbents were observed using a field emission scanning electron microscope (SEM) (S-4800, Hitachi, Japan). The As elemental mapping images were acquired using energy-dispersive X-ray spectroscopy (EDX) module coupled with the SEM under an electron accelerating voltage of 20 kV. The x-ray diffraction patterns (XRD) were acquired using a powder diffractometer with Cu Kα radiation (λ = 1.5418 Å) (D2 PHASER, AXS, Germany). The elemental valence and abundance analysis was conducted by using an X-ray photoelectron Spectrometry (XPS).

## Results and discussion

### Performance for arsenic removal

**Fig. 1:**
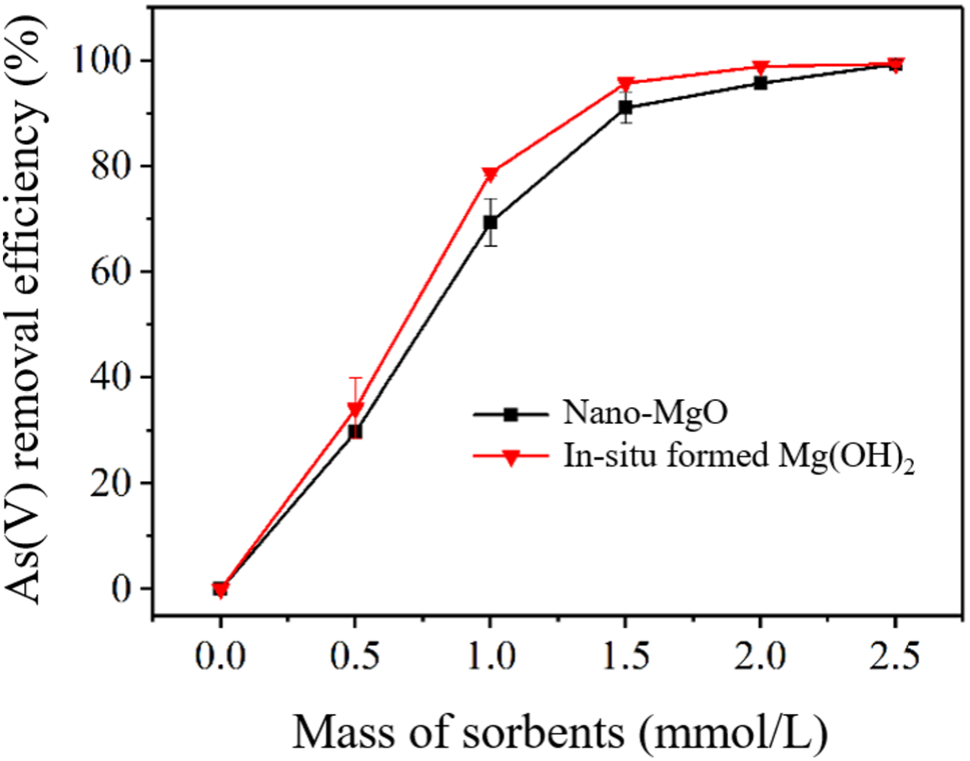
Comparison of the performance for As(V) removal by nano-MgO and Mg(OH)_2_-*in-situ* (initial As(V) = 10 mg/L, time = 24 h).

The adsorption performance of nano-MgO and *in-situ* formed Mg(OH)_2_ were compared by adding them into a simulated As(V) contaminated wastewater and analyzing the As(V) removal efficiencies. The results indicate that *in-situ* formed Mg(OH)_2_ and nano-MgO realized similar removal efficiencies while *in-situ* formed Mg(OH)_2_ realized slightly higher As(V) removal efficiency (Fig. 1). As the concentration of nano-MgO increased from 0.5 to 1.5 mmol/L, As(V) removal efficiency increased from 29.7% to 91.0%, while that of using *in-situ* formed Mg(OH)_2_ increased from 34.2% to 95.7% at the same application levels. In other words, to obtain an As concentration less than 0.5 ppm, which is the municipal wastewater treatment standard in China (GB 8978–1996), more than 2.0 mmol/L of nano-MgO will be needed, but only 1.5 mmol/L of *in-situ* formed Mg(OH)_2_ are needed. Nano-MgO has been proved to possess high adsorption capacity for both organic and inorganic As^10,23^. Thus, these results indicate that *in-situ* formed Mg(OH)_2_ from cheap MgCl_2_ and NaOH has similar or even better As(V)-removal capability than pre-formed nano-MgO with a much higher price.

**Fig. 2:**
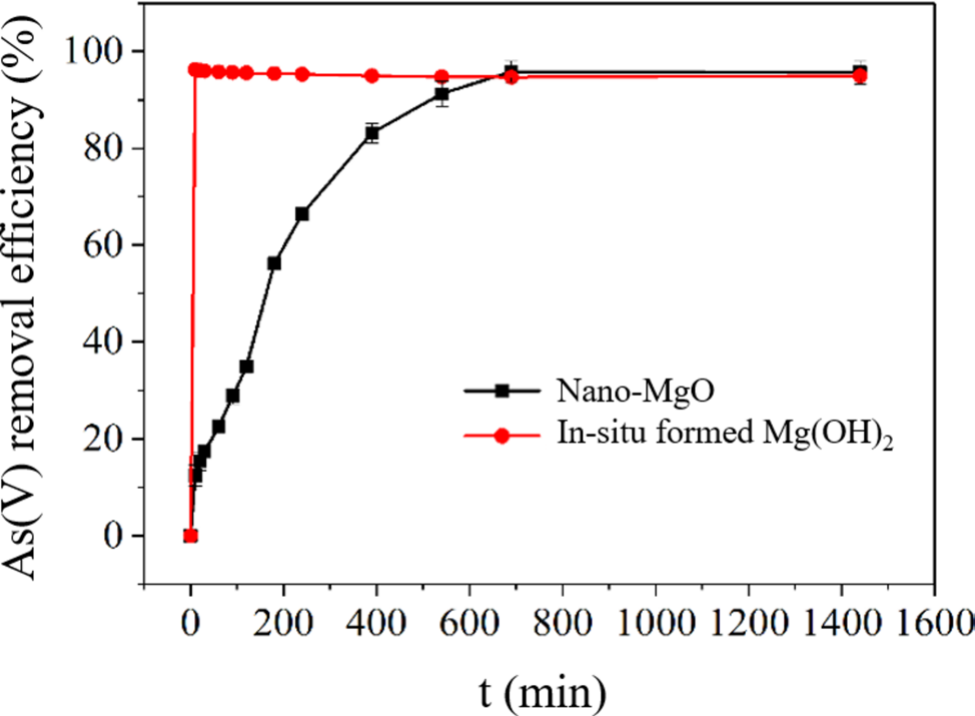
Comparison of As(V) removal kinetics between nano-MgO and Mg(OH)_2_-*in-situ* (initial As(V) = 10 mg/L, chemical dosage = 2 mmol/L).

The adsorption kinetics of As(V) on nano-MgO and *in-situ* formed Mg(OH)_2_ were studied, as illustrated in Fig. 2. A removal efficiency growth and equilibrium stages were observed for both chemicals, especially for *in-situ* formed Mg(OH)_2_. Specifically, for *in-situ* formed Mg(OH)_2_, the growth stage was extremely fast and finished within only 10 min, and nearly 99% of As(V) in water was adsorbed. This may be attributed to the abundant active binding sites of *in-situ* formed Mg(OH)_2_. Because As(V) could be efficiently incorporated into both the inner and outer surfaces of *in-situ* formed Mg(OH)_2_ flocs during the progress of flocs growth. Similar phenomenon was observed in adsorption of As(III) by *in-situ* formed Ti(OH)_4_^24^. On the other hand, the As(V) removal efficiency of nano-MgO, in contrast to that of *in-situ* formed Mg(OH)_2_, increased very slowly in the growth stage. It took as long as ~ 12 h to reach As(V) adsorption equilibrium. A possible reason might be that As(V) is removed during the phase transition of MgO into Mg(OH)_2_, which is a heterogeneous reaction between MgO and water, and will take longer time than a homogenous reaction between ions in water^23^. In addition, for the *in-situ* formed Mg(OH)_2_ treatment, the formed flocs settled down to the bottom very quickly within 30 min. This will facilitate efficient slurry separation via traditional gravity settling. Based on the above results, 10 min was chosen as the reaction time for the following experiments considering operational convenience and As(V)-removal efficiency. It should be noted that *in-situ* formed Mg(OH)_2_ didn’t realized higher As(V) removal efficiency but only accelerated the removal process. Thus, this process can’t be explained by a co-precipitation mechanism, which is process-controlled.

### Influence factors on arsenic removal by ***in-situ*** formed Mg(OH)_2_

Usually, pH was a key factor during adsorption of As(V). On the one hand, pH value affects As species in water according to their dissociation constants, which will influence the adsorption capacities of adsorbents^29^. On the other hand, pH value affects the formation of Mg(OH)_2_ which needs a slight basic pH condition^24^. In this study, As(V) removal by *in-situ* formed Mg(OH)_2_ was highly pH dependent and basic pH conditions could facilitate the As removal process (Figure S1). Differently, temperature had little influence on the As removal efficiency from 25 to 40 ℃, possibly due to the high reactivity of Mg^2+^ with OH^−^ (Figure S2).

**Fig. 3:**
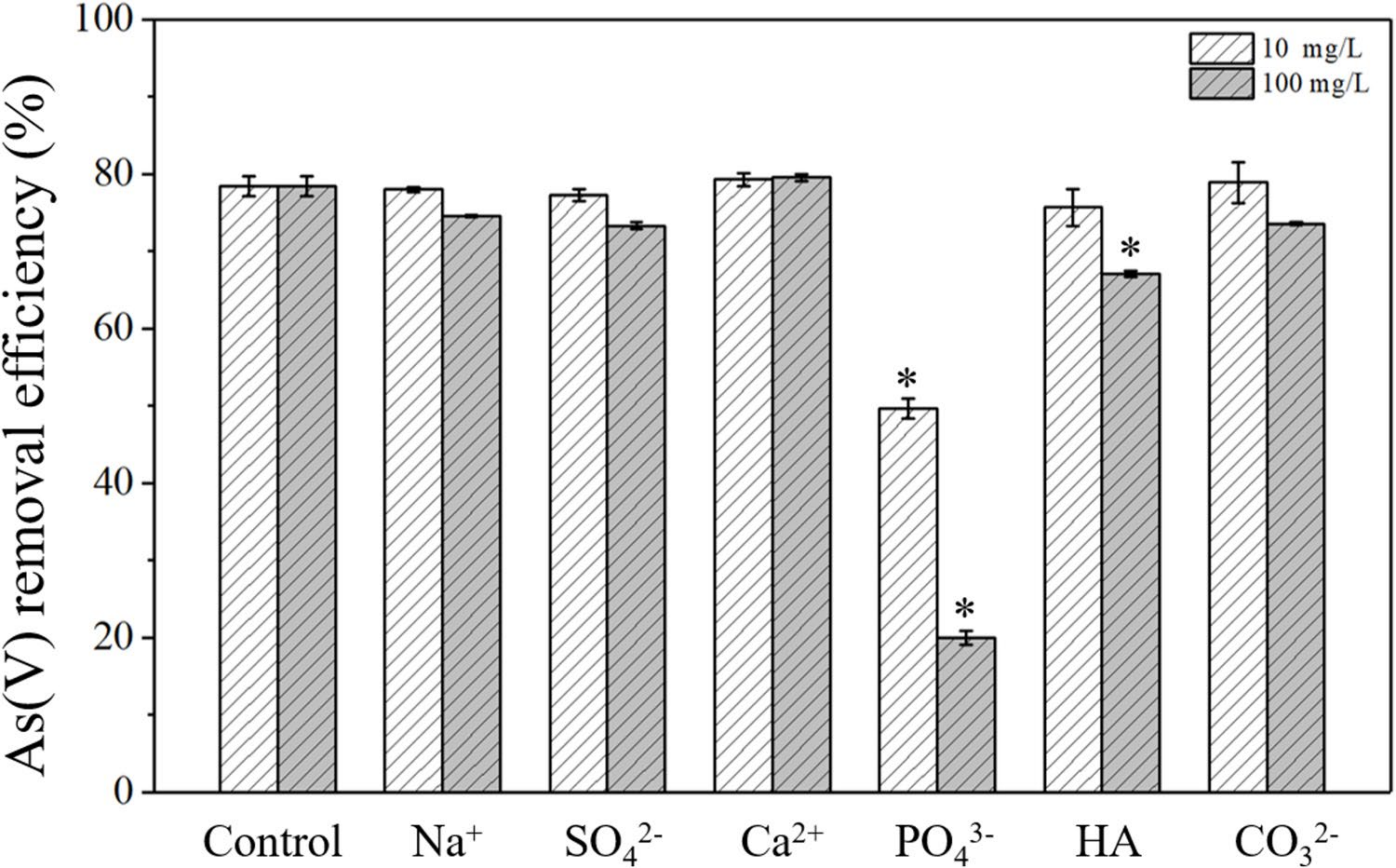
Effect of co-existed ions and HA on As(V) removal by *in-situ* formed Mg(OH)_2_ (initial As(V) = 10 mg/L, Mg(OH)_2_ = 1 mmol/L, time = 10 min). Here error bars mean standard deviation. A bar with an asterisk (*) represents significant difference from the control (p < 0.05).

Many ions co-exist with As in wastewater, such as Na^+^, Ca^2+^, and PO_4_^3−^. Moreover, HA, a common natural organic matter, was frequently reported to affect As removal^29–32^. Therefore, it is imperative to consider the presence of coexisting cations, anions, and HA while evaluating As(V) sorption capacity of *in-situ* formed Mg(OH)_2_. Here, two kinds of cation, Na^+^ and Ca^2+^, three kinds of anion, SO_4_^2−^, CO_3_^2−^, and PO_4_^3−^, and HA were chosen to investigate the effect of co-existed ions and HA on As(V) removal by *in-situ* formed Mg(OH)_2_. As illustrated in Fig. 3, Na^+^, Ca^2+^, CO_3_^2−^, and SO_4_^2−^ had slight or negligible influence on As(V) removal even at a concentration up to 100 mg/L.

Humic acid showed a slight negative effect on As(V) removal at high concentration of 100 mg/L, but no negative effect was observed at low concentration of 10 mg/L. The resistance to HA is an advantage of using *in-situ* formed Mg(OH)_2_. It was reported that the As(V) removal efficiency of an iron-cerium bimetal oxide material significantly decreased from 96.19% to 56.12% at the presence of only 10 mg/L HA^29^. Preformed sorbents are ease to encounter HA contamination on the material-water interface and lose their advanced adsorption property^33^. In this study, *in-situ* formed Mg(OH)_2_ has renewed material-water interface along with the *in-situ* formation of Mg(OH)_2_ so that the adsorption property could be retained^25,27^.

In contrast, PO_4_^3−^ was found to inhibit As(V) removal significantly with decreased efficiencies by 28.3% and 58.4% when encountering the interference of 10 and 100 mg/L of PO_4_^3−^, respectively. This is consistent with existing reports^1,29^. It is known than P and As are congeners in the periodic table and have similar chemical properties. PO_4_^3−^ acts as a competitive ion of AsO_4_^3−^ during As(V) removal using *in-situ* formed Mg(OH)_2_^34^.

### Properties of the formed Mg-As sludge by ***in-situ*** formed Mg(OH)_2_

**Fig. 4:**
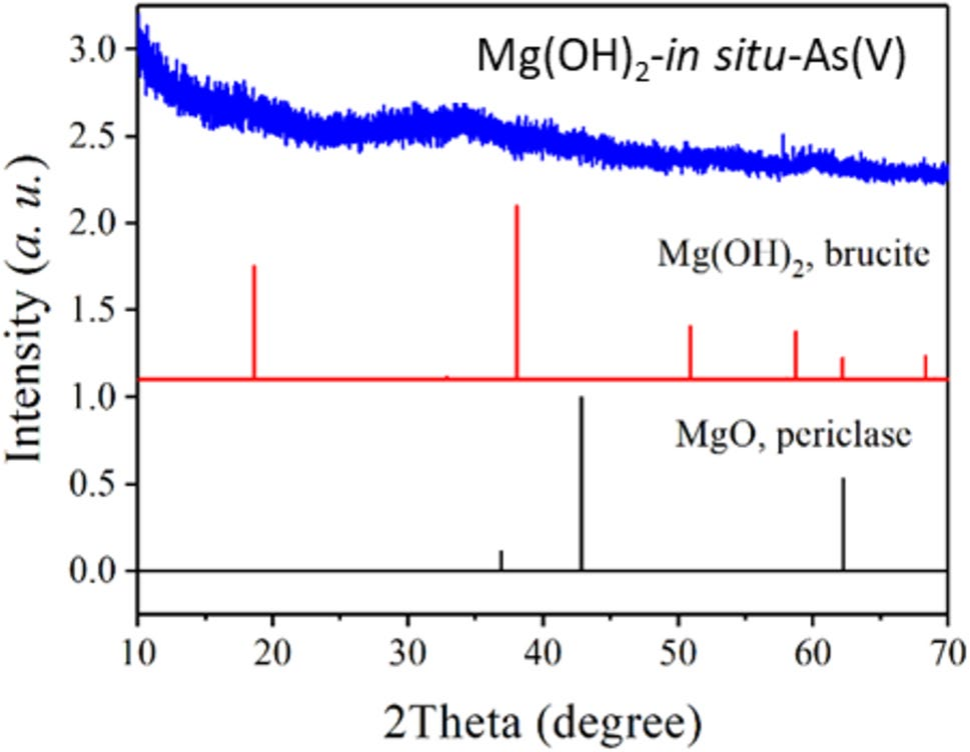
XRD image of MgO, Mg(OH)_2_ and the As(V)-loaded Mg(OH)_2_-in situ-As(V).

To explore the mechanism of the superior As(V) removal performance of *in-situ* formed Mg(OH)_2_, a series of characterizations were conducted on *in-situ* formed Mg(OH)_2_ flocs after As(V) adsorption. Firstly, the crystal structure of the *in-situ* formed Mg(OH)_2_ was characterized using XRD (Fig. 4). Obviously, there was no crystalline MgO or Mg(OH)_2_ in the *in-situ* formed Mg(OH)_2_ flocs compared to the standard XRD patterns of crystalline MgO (periclase) and Mg(OH)_2_ (brucite). Actually, no apparent diffraction peaks was observed in the flocs, indicating that the *in-situ* formed Mg(OH)_2_ existed as an amorphous form. Amorphous metal hydroxides were frequently reported with bigger specific surface areas, more surface hydroxy groups, and thus better adsorption performance^35^. Moreover, no apparent diffraction peaks were found for As minerals, indicating that As possibly existed as adsorbed ions. These results are consistent with existing reports on As coagulation by *in-situ* formed Ti(OH)_4_ and Fe(OH)_3_^24,25^.

**Fig. 5:**
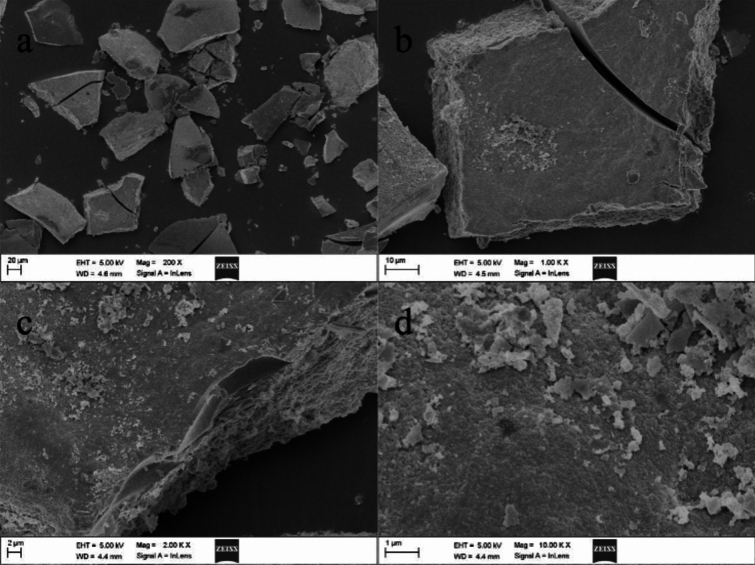
SEM image of the Mg(OH)_2_-in situ-As(V) sludge at different magnifications.

Micro-morphological results showed that the *in-situ* formed Mg(OH)_2_-As(V) sludge had a smooth bulk outlook (Fig. 5a). However, large numbers of wrinkles was seen on the surface and side at higher magnification (Fig. 5, b-d), indicating that *in-situ* formed Mg(OH)_2_ possessed a loose structure, which is consistent with its XRD pattern. Notably, the loose amorphous structure of *in-situ* formed Mg(OH)_2_ is completely different from the nanosheet-like Mg(OH)_2_ formed from nano-MgO hydrolysis^23^. This may be one of the reasons why *in-situ* formed Mg(OH)_2_ has superior As(V) removal performance, as an amorphous structure usually indicates a bigger specific surface area and more As binding sites.

**Fig. 6:**
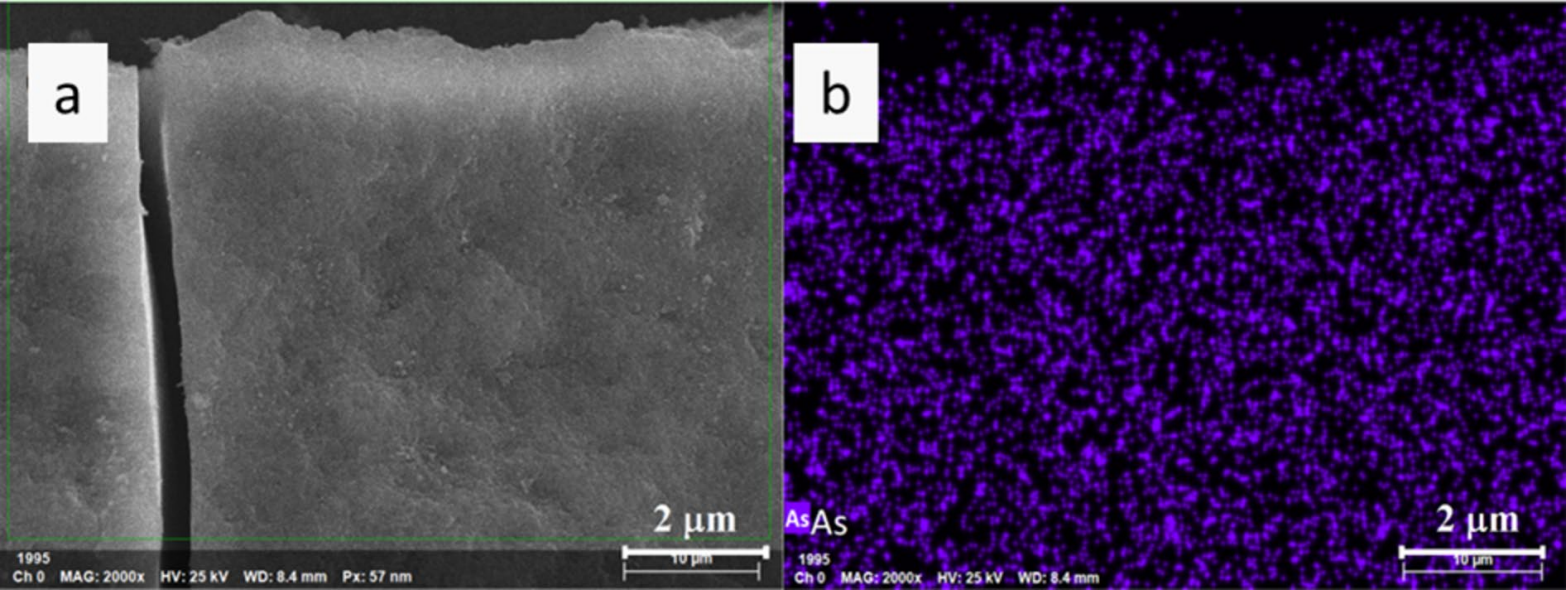
Elemental mapping of As in the Mg(OH)_2_-in situ-As(V) sludge. (**a**) SEM picture, (**b**) As distribution.

The elemental mapping picture indicates that the removed As(V) element distributed uniformly within the Mg-As(V) sludge (Fig. 6). Thus, As(V) was highly possible encapsulated within the precipitate, rather than adsorbed on the particle surface. It is a common sense that adsorption occurs on sorbent-water interface and is partially or completely reversible. The encapsulation might be the main reason about the high and fast As removal efficiency, which could transform reversible superficial adsorption into irreversible encapsulated co-precipitation and thus alleviate the desorption or second release of adsorbed As(V) back into water phase.

**Fig. 7:**
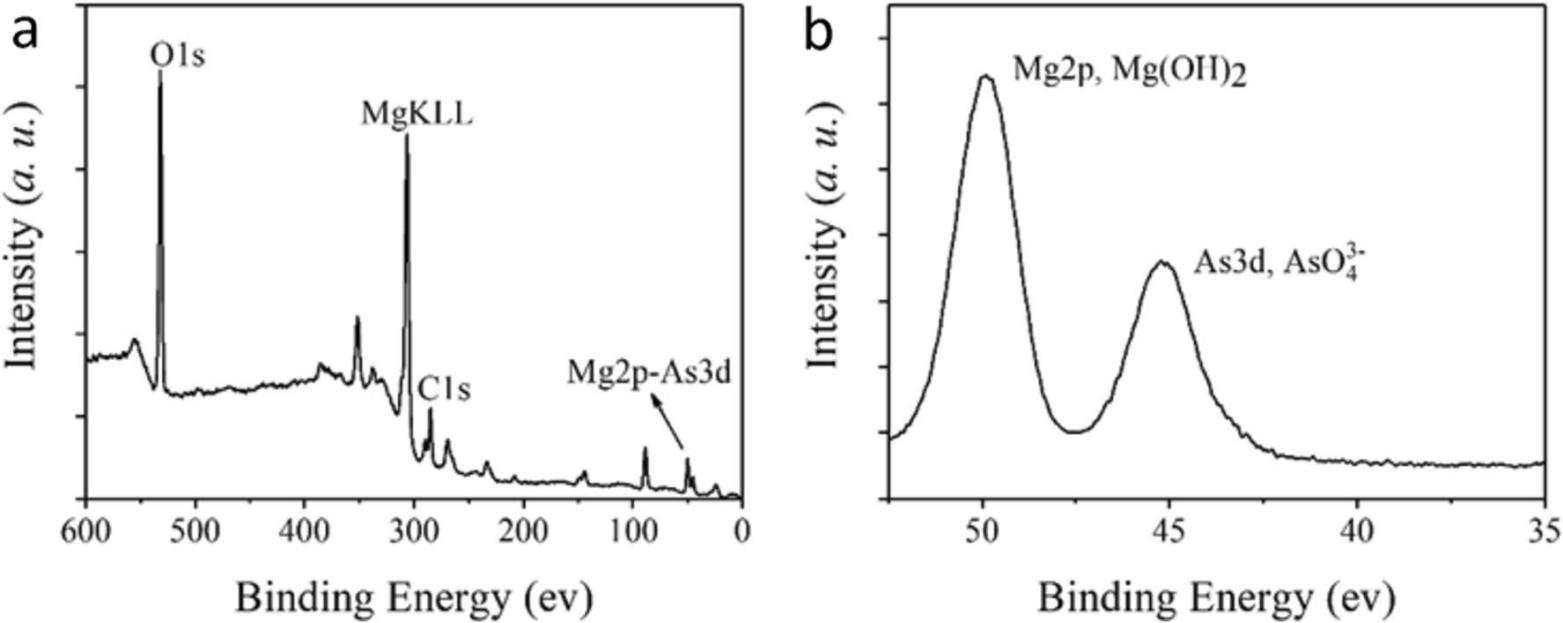
XPS analysis of the as-formed Mg(OH)_2_-in situ-As(V) sludge. (**a**) Full spectrum, (**b**) refined spectrum of Mg and As.

FTIR analysis was used to identify the main functional groups of *in-situ* formed Mg(OH)_2_ after As(V) adsorption. As shown in Figure S3, compared to MgO and Mg(OH)_2_ without As(V) exposure, there was an absorption peak appeared at 841 cm^−1^ in *in-situ* formed Mg(OH)_2_ after reacting with As(V). This can be attributed to the stretching vibration of As-O bonds, indicating that As (V) was transferred from the simulated wastewater to the newly generated precipitate. XPS was used to analyze the elemental composition and chemical valence of Mg and As in the flocs. According to the full spectrum, the flocs mainly consisted of Mg, O, and As (Fig. 7a). The refined Mg 2p and As 3d electron spectra indicate that Mg existed as Mg(OH)_2_ (49.89 eV) and As existed as AsO_4_^3−^ (45.01 eV) (Fig. 7b). The comparable peak intensity indicates high As content in the formed Mg-As sludge.

**Fig. 8:**
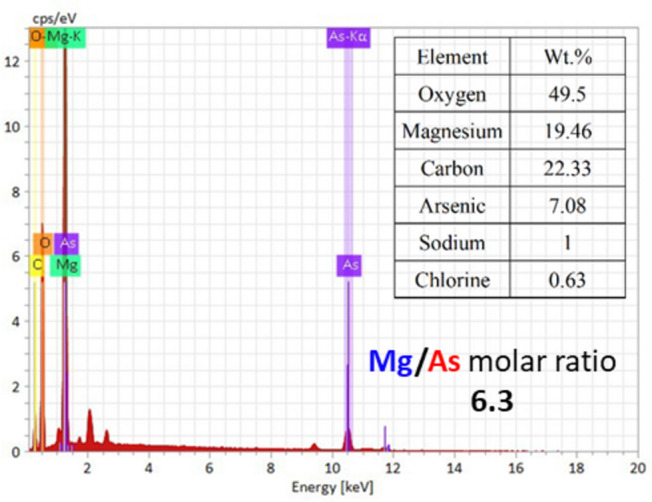
EDX spectrum image of the Mg(OH)_2_-in situ-As(V) sludge.

EDX reveals a very low Mg/As molar ratio of ~ 6.3 (Fig. 8). These result indicates a very high atomic utilization efficiency of Mg^2+^ during the As(V) removal process, which could hardly be achieved by using MgO nanoparticles. As(V) was quite possibly encapsulated uniformly within amorphous Mg(OH)_2_ via coordination rather than forming magnesium arsenate crystal. Similarly, no magnesium arsenate was observed in As(V) removal by preformed commercial Mg(OH)_2_^21^. The high Mg atomic utilization efficiency implies a low cost and highly reduced mass amount of Mg-As sludge. Therefore, the post-treatment of the hazardous As-sludge will be easier.

### Mechanism of As removal using in-situ formed Mg(OH)_2_

**Fig. 9:**
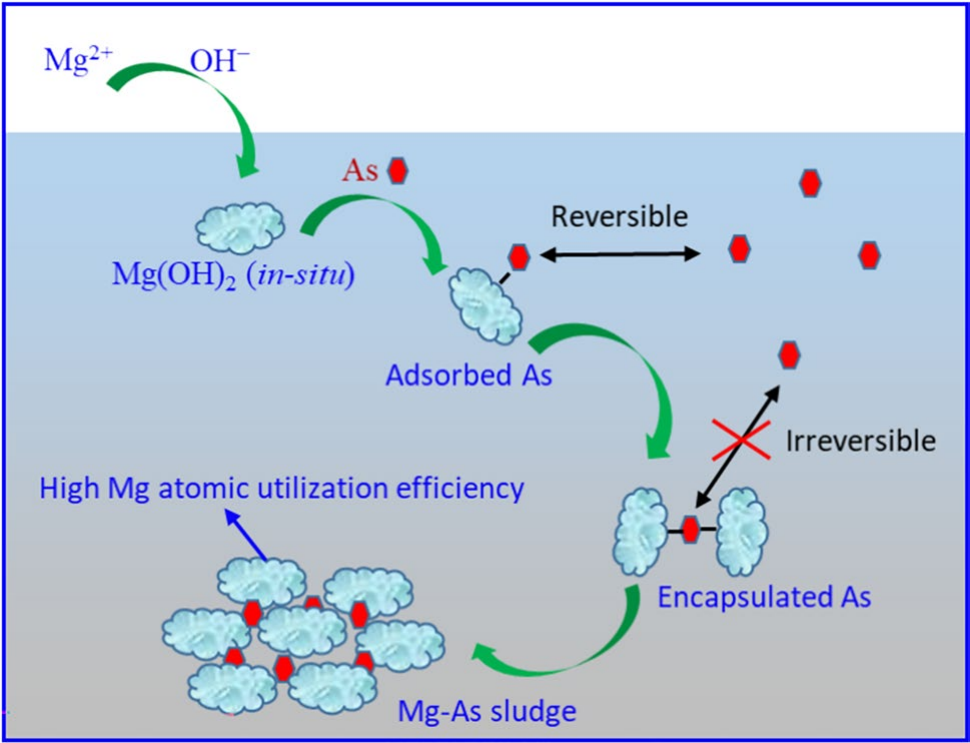
The possible mechanism for the enhanced As(V) removal from water by Mg(OH)_2_-in situ.

Based on the above results, a simplified mechanism is proposed and shown in Fig. 9. During the successive addition of MgCl_2_ and NaOH solutions to As(V) contaminated wastewater, amorphous Mg(OH)_2_ tiny nuclei (Mg(OH)_2_(*in-situ*)) are formed with high affinity surface hydroxyl groups and big surface area. Simultaneously, As(V) is adsorbed onto the Mg(OH)_2_ surface. Then, the nuclei collide with each other and form bigger flocs with As(V) encapsulated within the flocs. Ultimately, Mg-As(V) composite flocs settle down to bottom and As(V) is removed from water. As the Mg(OH)_2_ sorbent is *in-situ* formed within the As(V) contaminated water, tiny Mg(OH)_2_ nuclei are formed with renewed Mg(OH)_2_-water interface from basic Mg^2+^ and OH^−^ ions with very abundant adsorption active sites. Meanwhile, the adsorbed As(V) hinders the further growth of Mg(OH)_2_ nuclei. Thus, amorphous Mg-As sludge is formed instead of Mg(OH)_2_ nanocrystals.

## Conclusions

Efficient As(V) removal from water is realized by using *in-situ* formed Mg(OH)_2_, which is obtained by simply adding cheap MgCl_2_ and NaOH reagents. Notably, the removal process is very quick and can be performed within several minutes while traditional methods using MgO nanoparticles need several hours. This process is not only timely efficient but also cost-effective than using MgO nanoparticles as sorbent. Further, the amount of the resulted Mg-As sludge can be reduced significantly and thus adapt better to the demand of mass reduction on hazardous waste disposal. The mechanism still needs further study and a possible explanation is that *in-situ* formed Mg(OH)_2_ has more accessible and high affinity surface hydroxyl groups because of the precipitation reaction starting from basic Mg^2+^ and OH^−^ ions. As(V) is incorporated into the inner surface of *in-situ* formed Mg(OH)_2_ agglomerates, hinders its crystallization process, and finally forms an amorphous Mg-As precipitate. Although the feasibility in treating real wastewater in large scale is still unclear, considering the very fast, efficient As(V) removal process and low cost of MgCl_2_ and NaOH reagents, this As removal technology based on *in-situ* formed Mg(OH)_2_ has great potential for scale application and will imply a bunch of other *in-situ* formed nanomaterials for water treatment.

## Supplementary Information

Supplementary Information.

## Data Availability

Data is provided within the manuscript or supplementary information files.
