# Supplementary material for: Efficient and fast arsenate removal from water by in-situ formed magnesium hydroxide
Source: Sci Rep. 2024 Sep 11;14:21232. doi: 10.1038/s41598-024-72258-6 (PMC11390963; doi:10.1038/s41598-024-72258-6)
Supplement: Supplementary file 1 — Supplementary Information. [file 41598_2024_72258_MOESM1_ESM.docx]

**Supplementary Information**

**Efficient and fast arsenate removal from water by** ***in-situ* formed magnesium hydroxide**

Juanjuan Zhou ^a^, Ying Yang ^b^, Zhanjun Li ^b, c^ *

^a^ School of Health, Guangzhou Vocational University of Science and Technology, Guangzhou, 510080, P.R. China

^b^ School of Environment, Jinan University, Guangzhou, 511436, P.R. China

^c^ School of Biomedical Engineering, Guangzhou Medical University, Guangzhou, 511436, P.R. China

*Corresponding author: Zhanjun Li, email: zhanjunli@gzhmu.edu.cn


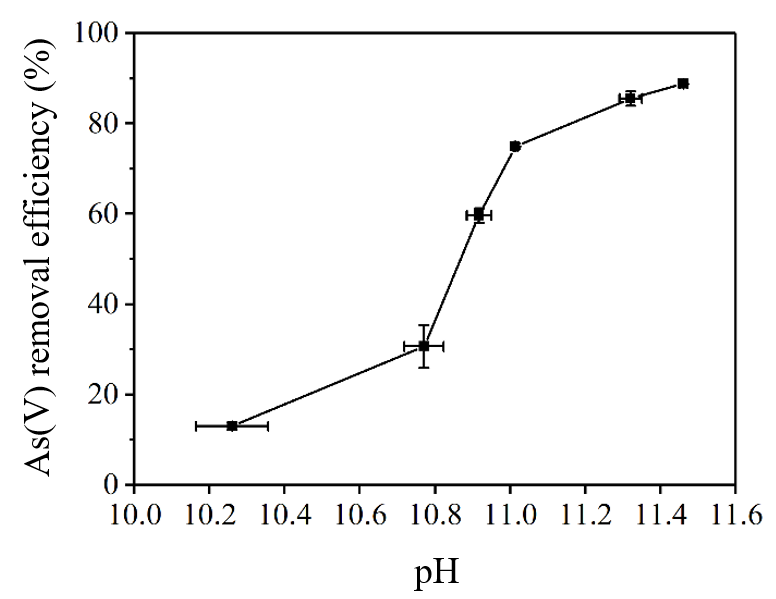


**Figure S1.** Effect of pH on As(V) removal by *in-situ* formed Mg(OH)_2_ (initial As(V) = 10 mg/L, Mg(OH)_2_ = 1 mmol/L, time = 10 min).


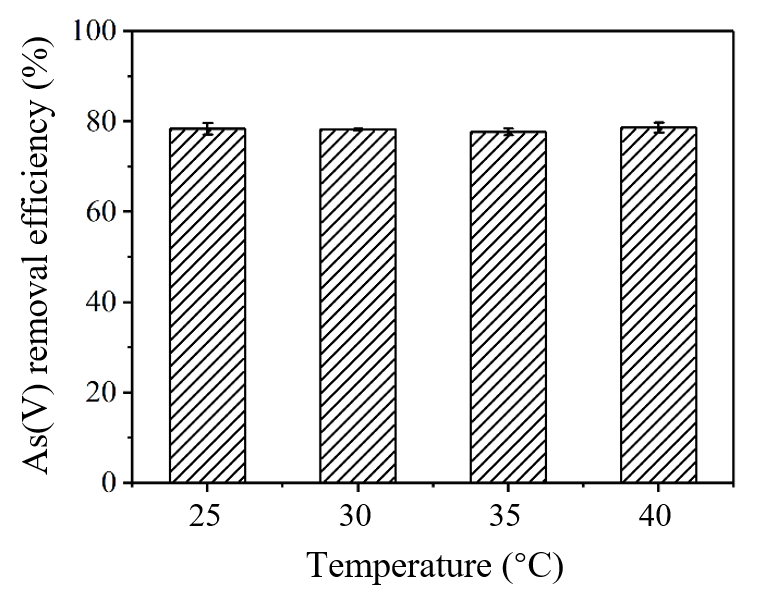


**Figure S2.** Effect of temperature on As(V) removal by *in-situ* formed Mg(OH)_2_ (initial As(V) = 10 mg/L, Mg(OH)_2_ = 1 mmol/L, time = 10 min).


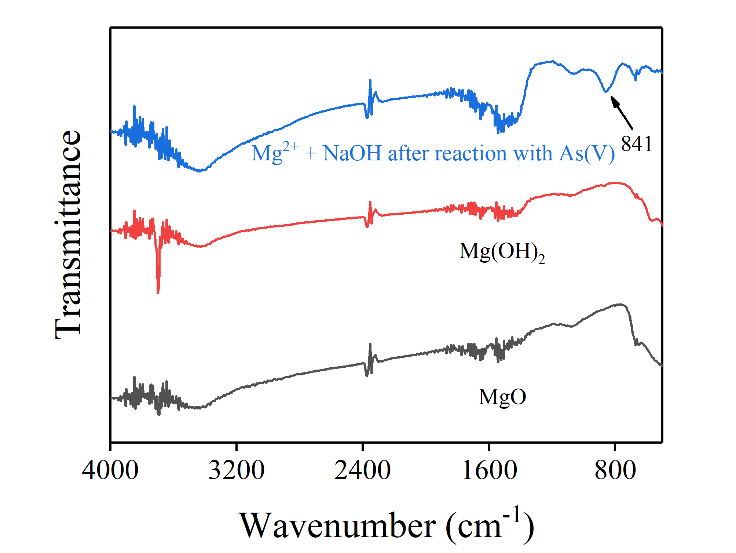


**Figure S3.** FTIR spectra image of MgO, Mg(OH)_2_ and the As(V)-loaded Mg(OH)_2_.
